# Crossover Control Study of the Effect of Personal Care Products Containing Triclosan on the Microbiome

**DOI:** 10.1128/mSphere.00056-15

**Published:** 2016-05-18

**Authors:** Angela C. Poole, Lauren Pischel, Catherine Ley, Gina Suh, Julia K. Goodrich, Thomas D. Haggerty, Ruth E. Ley, Julie Parsonnet

**Affiliations:** a Department of Molecular Biology and Genetics, Cornell University, Ithaca, New York, USA; b Department of Medicine, Stanford University School of Medicine, Stanford, California, USA; c Department of Microbiology, Cornell University, Ithaca, New York, USA; d Department of Health Research and Policy, Stanford University School of Medicine, Stanford, California, USA; Arizona State University

**Keywords:** triclosan, microbicide, microbiome, randomized double-blind crossover study

## Abstract

Commonly prescribed antibiotics are known to alter human microbiota. We hypothesized that triclosan and triclocarban, components of many household and personal care products (HPCPs), may alter the oral and gut microbiota, with potential consequences for metabolic function and weight. In a double-blind, randomized, crossover study, participants were given triclosan- and triclocarban (TCS)-containing or non-triclosan/triclocarban (nTCS)-containing HPCPs for 4 months and then switched to the other products for an additional 4 months. Blood, stool, gingival plaque, and urine samples and weight data were obtained at baseline and at regular intervals throughout the study period. Blood samples were analyzed for metabolic and endocrine markers and urine samples for triclosan. The microbiome in stool and oral samples was then analyzed. Although there was a significant difference in the amount of triclosan in the urine between the TCS and nTCS phases, no differences were found in microbiome composition, metabolic or endocrine markers, or weight. Though this study was limited by the small sample size and imprecise administration of HPCPs, triclosan at physiologic levels from exposure to HPCPs does not appear to have a significant or important impact on human oral or gut microbiome structure or on a panel of metabolic markers.

**IMPORTANCE** Triclosan and triclocarban are commonly used commercial microbicides found in toothpastes and soaps. It is unknown what effects these chemicals have on the human microbiome or on endocrine function. From this randomized crossover study, it appears that routine personal care use of triclosan and triclocarban neither exerts a major influence on microbial communities in the gut and mouth nor alters markers of endocrine function in humans.

## Abstract (precis)

Triclosan and triclocarban are commonly used commercial microbicides found in toothpastes and soaps. It is unknown what effects these chemicals have on the human microbiome or on endocrine function. From this randomized crossover study, it appears that routine personal care use of triclosan and triclocarban neither exerts a major influence on microbial communities in the gut and mouth nor alters markers of endocrine function in humans.

## INTRODUCTION

Triclosan [5-chloro-2-(2,4-dichlorophenoxy)phenol] and triclocarban [3-(4-chlorophenyl)-1-(3,4-dichlorophenyl)urea] (TCS) are broad-spectrum phenolic biocides with activity against both bacteria and fungi. First licensed for use in the United States in the late 1950s (triclocarban) and early 1960s (triclosan), TCS have rapidly become nearly ubiquitous in exposures for humans. TCS have been integrated or impregnated into a wide range of household cleaning and personal care products (HPCPs) ranging from soaps to clothing and children’s toys. In the environment, triclosan has been detected in 50% of surface water samples from areas where adverse effects on aquatic microbiota have been of concern (1–3). As recently as 2008, triclosan was so widely employed that it was identified in 75% of human urine samples in the United States (4).

Although toothpaste with triclosan reduces plaque and gingivitis, no other benefits to human health have been established to support the use of TCS in other HPCPs (5). In *in vitro* systems and animal models, triclosan has been reported to be an endocrine disruptor, mimicking or altering thyroid hormone, estrogen, and testosterone function (6–10). In addition, there have been concerns that widespread TCS use may contribute to antimicrobial resistance (11). Because of the lack of proven efficacy and concerns about systemic effects, TCS are currently under scrutiny by U.S. and European regulatory agencies. In December of 2013, the FDA issued a “proposed rule” requiring manufacturers of antibacterial hand soaps and body washes to provide evidence within 1 year that their TCS products are both safe and more effective than plain soaps in preventing illness (12). In response to both governmental and public concerns, TCS have now been largely removed from commercial soaps in the United States. However, triclosan remains in some toothpaste and hospital products, and triclocarban is still incorporated in nonconsumable household items.

The effects of broad exposure to TCS on healthy human microbiota have not been thoroughly addressed. Changes in the composition of the microbiome have been implicated as a causal factor in common diseases, including diabetes, metabolic syndrome, and obesity (13–17). Commonly prescribed antibiotics are known to perturb the microbiome (18). Moreover, long-term, low-dose exposure to antibiotics has been hypothesized to be a driver in the obesity epidemic (19). Low-dose antibiotic treatment early in life has also been associated with increased weight gain in farm animals and mice, an effect thought to be partially mediated by the gut microbiome (20–23). Although no comprehensive studies of the effects of triclosan on the healthy human oral microbiome have been published, triclosan is known to mitigate plaque burden and gingivitis (5). With the goal of understanding the effects of TCS on the composition and diversity of the microbiome and host health, we conducted a double-blind, randomized, crossover study of TCS-containing HPCPs in adult volunteers and assessed effects of TCS on both oral and gut microbiome compositions and endocrine function.

## RESULTS

### Triclosan.

**FIG 1:**
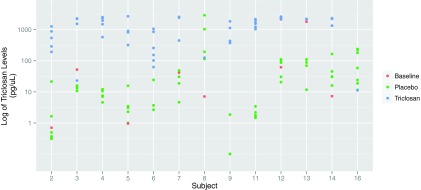
Triclosan levels in the subjects. Urine samples were tested in triplicate for levels of both triclosan and glucuronidated triclosan, and 97% of the samples were used in the analysis (one sample was excluded due to technical problems, and three were excluded due to date of collection [those samples were taken 1 day after switching between arms]). Seventy-one subsamples far exceeded the upper limit of quantification (>70 pmol/µl), and 84 subsamples were below 10 fmol/µl; 47 (35%) samples were affected by this rounding.

As expected, urinary triclosan levels were higher in the TCS phase (median, 1,548 pg/µl) than in the non-TCS (nTCS) phase (median, 14.6 pg/µl) (*P* < 0.001) (Table 1 and Fig. 1). In the TCS phase, triclosan was detected in 100% of samples, whereas in the nTCS phase, levels were below the limits of detection in 28% of nTCS samples. One subject (subject 8) had markedly higher levels of triclosan in the nTCS phase (616 pg/µl on average) than in the TCS phase **(**125 pg/µl). Another subject (subject 16) provided urine only once, while in the TCS phase; that triclosan level was lower than the mean of the placebo-phase levels (11 pg/µl versus 58 pg/µl, respectively).

**TABLE 1:** Changes in selected metabolic and endocrine markers across the study^a^

Marker	Value(s)			
	Median (Q1–Q3) at baseline	Median at end of TCS phase	Median at end of nTCS phase	*P*
Triclosan urine (pg/µl)	24.4 (7.2–59.4)	1,548	14.6	<0.001
CRP (ng/ml)	244 (146–406)	178	238	0.65
Free testosterone (ng/dl)	0.6 (0.4–8)	0.4	0.55	0.16
GIP (pg/ml)	437 (328–766)	277	281	0.53
Insulin (pg/ml)	208 (113–295)	226	223	0.81
Leptin (ng/ml)^b^	3.6 (1.9–5.4)	3.8	4.5	0.15
Triglycerides (mg/dl)	70 (53–91)	84	70	0.39
TSH (mIU/liter)	1.4 (0.9–1.6)	1.24	1.5	0.18

a Q1–Q3, quintile 1 to quintile 3; TCS, triclosan products; nTCS, nontriclosan products; CRP, C-reactive protein; GIP, gastric inhibitory polypeptide; TSH, thyroid-stimulating hormone.

b Biomarker measured twice; results combined.

### Weight and serum biomarkers.

Subjects in this study were generally of normal weight (Table 2). Six subjects gained more than 0.6% of body weight (the highest quartile) in the TCS phase but lost weight or stayed at the same weight in the nTCS phase. The odds ratio (OR) for gaining weight in the TCS phase, compared to losing weight or showing no change in weight, was 5 (95% confidence interval, 0.6 to 236; *P* value, 0.22).

**TABLE 2:** Characteristics of study subjects

Parameter	Value(s)		
	Baseline	Period 1	Period 2
No. of subjects	16	14	13
Mean age (yrs)	43	43	44
No. (%) of females	11 (73)	11 (79)	10 (77)
No. (%) of while, non-Hispanic subjects	11 (73)	10 (71)	10 (77)
Median (25th–75th percentile) wt (kg)	65.4 (58.3–77.1)	65.3 (57.3–77.1)	66.1 (54.5–74.8)
No. (%) of subjects with TCS exposure		7 (50)	6 (46)

No significant differences by phase were found in levels of testosterone, T4, thyroid-stimulating hormone (TSH), or any of the 25 obesity and diabetes markers measured (Table 1; see also Table S1 in the supplemental material). When the analysis was repeated with one outlier removed (subject 8) (Fig. 1), leptin levels were 18% higher in the nTCS phase than in the TCS phase (*P* value, 0.02), but not after correction for multiple comparisons (Bonferroni method). No other laboratory measures were different between phases after removing the outlier.

10.1128/mSphere.00056-15.1 Table S1  Median values of remaining obesity and diabetes markers by phase. Download Table S1, DOCX file, 0.02 MB. Copyright © 2016 Poole et al. 2016 Poole et al. This content is distributed under the terms of the Creative Commons Attribution 4.0 International license.

### TCS effects on the microbiome.

**FIG 2:**
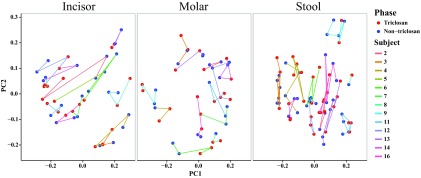
Microbial diversity is influenced by subject of origin, not by study phase. Each panel shows principal coordinate 1 (PC1) and PC2 data from principal-coordinate analysis (PCoA) of the unweighted Unifrac distances for the sample types indicated: incisor plaque, molar plaque, and stool. Each sphere represents a single sample collected from one individual and is colored according to the phase during which the sample was collected. All samples from each subject are connected by a line in the order collected, and the lines are colored by subject.

Microbiome analysis was conducted on those samples collected during the latter half of each period; these samples included a total of 3,592,186 sequences with a median sequence count of 81,633 sequences for the 45 molar gingival plaque samples, a total of 3,841,706 sequences with a median sequence count of 83,064 sequences for the 46 incisor gingival plaque samples, and a total of 6,124,500 sequences with a median sequence count of 71,830 for the 76 stool samples. Analyses were performed separately for each of the sample sites (molar plaque, incisor plaque, and stool). For each site, principal-coordinate analysis (PCoA) of the unweighted Unifrac distance metric matrix showed clustering of samples based on the individual sampled rather than on the treatment phase, indicating that the principal driver of the diversity differences between samples was interindividual differences in microbiomes (Fig. 2).

A linear model with mixed effects was used to determine whether or not TCS exposure affected the relative abundances of particular taxa. Prior to multiple-testing correction, we observed significantly lower relative abundances of several taxa, including *Lautropia* in the molar gingival plaque samples and *Prevotella*, *Fusobacterium*, and *Veillonella* in the incisor gingival plaque samples in the TCS phase (Table 3). Several members of these genera have been previously associated with gingivitis and/or periodontitis (24–29). In the stool samples, the levels of bacteria of the genus *Bacteroides*, known to ferment polysaccharides, were decreased in the TCS phase (30). However, none of these associations passed false-discovery-rate (FDR) correction. Furthermore, there were no significant differences in the alpha diversities (species richness) of the microbiomes between the TCS and nTCS phases for any of the three sites. Results did not meaningfully differ when subject 8, who had higher triclosan levels in the nTCS phase than in the TCS phase, was removed from the analysis.

**TABLE 3:** Differentially abundant taxa in oral and gut microbiomes

Classification of taxon	Sample type	*P* value	Adjusted *P* value	Abundance in triclosan phase relative to nontriclosan phase
*Proteobacteria*; *Lautropia*	Molar	0.006	0.54	Decreased
TM7; unclassified TM7-3	Molar	0.025	0.59	Increased
*Firmicutes*; unclassified *Bacillaceae*	Molar	0.039	0.59	Decreased
*Firmicutes*; unclassified *Streptococcaceae*	Molar	0.040	0.59	Decreased
*Firmicutes*; *Moryella*	Molar	0.040	0.59	Increased
*Proteobacteria*; *Acinetobacter*	Molar	0.042	0.59	Increased
*Firmicutes*; unclassified *Carnobacteriaceae*	Incisor	0.012	0.42	Decreased
*Bacteroidetes*; *Prevotella*	Incisor	0.012	0.42	Decreased
*Fusobacteria*; *Fusobacterium*	Incisor	0.016	0.42	Decreased
*Firmicutes*; *Gemellales*	Incisor	0.018	0.42	Decreased
*Actinobacteria*; *Rothia*	Incisor	0.024	0.45	Increased
*Firmicutes*; *Veillonella*	Incisor	0.039	0.59	Decreased
*Bacteroidetes*; *Bacteroides*	Stool	0.012	0.93	Decreased
*Firmicutes*; *Leuconostoc*	Stool	0.025	0.93	Decreased
*Firmicutes*; *Megasphaera*	Stool	0.033	0.93	Increased
*Bacteroidetes*; unclassified *Bacteroidales*	Stool	0.040	0.93	Decreased
*Firmicutes*; *Dehalobacterium*	Stool	0.043	0.93	Increased

## DISCUSSION

For this double-blind, randomized crossover study, we hypothesized that oral microbiome composition would be altered by repeated, direct exposure to a triclosan-containing dentifrice and that gut microbiome composition would perhaps be affected as well due to systemic exposure to TCS via multiple products, as evidenced by detection of triclosan in the urine. However, in comparing microbial compositions between the TCS and nTCS phases, we observed neither a loss of species richness nor a shift in overall diversity. Although levels of a few taxa previously associated with oral diseases were reduced in the TCS phase, the differences in abundances between phases did not achieve significance in multiple testing. This lack of difference could have been a consequence of the fact that the subjects had been exposed to TCS for an extended period of time throughout their lives and their microbiota had already adapted to TCS exposure—a trend then not reversed with a lower exposure to TCS for several months. However, a washout period of at least 16 days prior to beginning the study was used in an attempt to control for this factor. Another possible reason for a lack of difference could be that the levels of TCS that were attained were not high enough. Overall, though TCS appears to both perturb environmental systems and have multiple toxicities in animals, it has not been shown to adversely impact human endocrine function health at levels achieved from commercial HPCPs (31, 32). In other circumstances, TCS does not appear to have the intended or expected antimicrobial effects (33). The differences in TCS effectiveness were likely due to the use of different concentration levels in different contexts (32, 33). However, though the levels might be low in the urine, they could have been much higher in the mouth, where toothpaste is directly applied. The oral flora was where we expected to find the greatest effect, but we still saw none.

These results, however, are consistent with the prior finding that the use of triclosan-containing toothpaste reduces the overall bacterial load (34). Thus, we would expect microbial biomass to be reduced in the TCS phase without a shift in microbial community composition. Although a study performed with more individuals could increase our power to detect differences in the levels of specific microbial taxa, major shifts in the levels of microbial flora from triclosan seem unlikely. In comparison to results from some other studies, where a small number of subjects was sufficient to allow detection of a significant difference in the microbiome (35), our null results indicate that a very large study population would be needed to identify any TCS-related alterations of the microbiome, if they do, in fact, exist.

We hypothesized that triclosan, either directly or through its antimicrobial effects, might alter host endocrine function and markers for obesity, diabetes, and inflammation. The triclosan levels obtained at baseline (picogram-per-microliter range), while somewhat lower than those observed in the NHANES study (nanogram-per-microliter range), were consistent with those found by other investigators (36, 37). For the 25 endocrine, obesity, and diabetes markers analyzed, none showed a significant difference or a clear trend toward a significant difference between TCS and nTCS phases. A difference in endocrine markers could have been explained either by an effect of triclosan or by disruption of the microbiome as a consequence of the activity of gastric inhibitory polypeptide (GIP) and glucagon-like peptide (GIP) (8, 38). Our results support the findings of other reassuring reports indicating that, though toxic to animals at high doses, TCS is not an endocrine disruptor in routine use in HPCPs in humans (36, 39). Finally, although inflammation and gingivitis have both been linked to metabolic syndrome (40), no changes in inflammatory markers were observed between the two study phases.

As expected, triclosan levels were significantly different between the TCS and nTCS phases. This supports the validity of the study by demonstrating that exposure to home care products was sufficient to alter triclosan levels. Surprisingly, one participant, subject 8, had higher triclosan values in the nTCS phase than in the TCS phase; however, this single value was measured at the beginning of the period, before the subject had had sustained exposure to TCS. Although it is possible that the subject mislabeled a sample or inadvertently used the wrong product during the nontriclosan period, unfortunately, such an error cannot be proven. Removing subject 8 from the analysis, however, did not change the results. Subject 16 also had higher levels of triclosan in the nontriclosan period than in the active phase. However, these levels were within the range of measurements taken from other subjects during the nontriclosan period. Furthermore, for this subject, only one value was taken in the TCS phase, and this value was lower than those seen among other study subjects.

Interestingly, there was a nonsignificant trend for participants to gain weight while in the TCS phase and to lose weight in the nTCS phase. Triclosan has been previously associated with an elevated body mass index (BMI) in the NHANES data set (37). Two subsequent studies have been contradictory on the matter, one reporting decreased BMI associated with increasing triclosan and another finding no association between triclosan levels and weight (41, 42). Our study was not adequately powered in that respect, and looking at changes in weight was not its primary objective. Given the inconclusive evidence surrounding triclosan and weight, these data may suggest an underlying correlation between increased triclosan levels and increasing BMI that is independent of changes in the microbiome or tested biomarkers but would require a larger study for verification.

This study was limited by the use of a small number of subjects, a complex sampling schedule for subjects to follow, and multiple comparisons. Further, there could have been great variability in the amounts and routes of triclosan exposure, as subjects were given a variety of HPCPs and not given precise instructions on how to use these products. This variability could have differently affected the gut and teeth microbiomes and might not directly correlate with the urine triclosan levels. Furthermore, although subjects also used triclocarban-containing bar soap while in the active phase, we did not measure triclocarban in urine samples. We are currently evaluating the effect of TCS on skin flora. Another potential source of misclassification bias is exposure to triclosan in the general community; this exposure may have contributed to the high levels of triclosan in four subjects during the control phase. Exposure to other, nonmeasured antimicrobials in the community such as parabens and nanosilver could also bias our findings toward the null. Finally, some of the obesity and diabetes panel tests returned indeterminate results, limiting the power of the study and the ability to explore trends.

In summary, though this study was limited by the small sample size and lack of specificity in product administration, triclosan at physiologic levels obtained from HPCPs did not have a major impact on human gut or oral microbiome compositions or on the series of metabolic and inflammatory markers assayed in this small study. Given the ubiquity of triclosan use over the last few decades, these results are reassuring. However, trends in our data indicate that triclosan may impact important members of the microbiome and decrease the total microbial biomass. Since triclosan remains a component of some of the most common HCPCs in the United States, we advocate for more focused research, particularly on oral flora, to assess whether taxonomic shifts subtler than those that we could observe could have long-term influences on human health.

## MATERIALS AND METHODS

### Recruitment.

Healthy volunteers from Stanford University were recruited from July 2011 to March 2013 using flyers, talks, and informal discussions with the goal of enrolling 12 to 20 participants. Inclusion criteria were age greater than 18 years and willingness to use study-assigned HPCPs (toothpaste, hard and liquid soap, dish soap) for 8 months. All recruitment and follow-up occurred prior to the FDA-proposed rule, and many of the subjects regularly used TCS-containing HPCPs in their homes. Exclusion criteria included recent use of antibiotics, gastrointestinal illness or travel to a developing country within the prior 3 months, pregnancy, unwillingness to change personal hygiene products, and high likelihood of noncompliance. Upon completion, subjects received a $200 gift card. The study was approved by the Institutional Review Board of Stanford University (ClinicalTrials.gov identifier NCT01509976).

### Enrollment and randomization.

**FIG 3:**
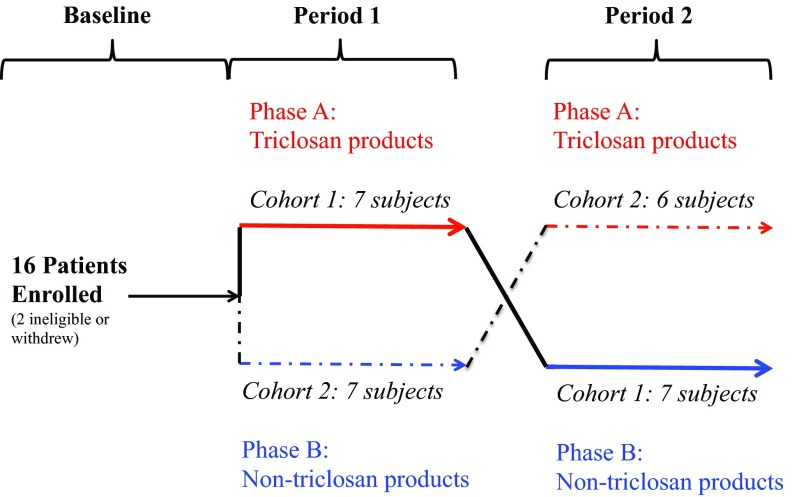
Graphic of enrollment, randomization, and study progression. The two potential paths for each subject are shown by solid and dashed lines. Phase data indicate whether the subject was using TCS-containing or non-TCS-containing products. Period data indicate the first or second 4-month period in the study for that participant. Cohort data indicate the subjects who received TCS or nTCS products in a specific order. Numbers of subjects in each cohort are indicated by period.

At enrollment, participants provided informed consent and were randomized to either the TCS phase (phase A; red labels) or the non-TCS phase (phase B; blue labels) (Fig. 3). HPCPs included toothpaste, liquid hand soap, solid hand soap, and dishwashing liquid. Commercially available products were deidentified with respect to company and product information. Subjects then started a washout period of at least 16 days in which they removed all TCS-containing products from their house and then remained in each phase for 4 months. No specific instructions were given on how frequently the subjects were to use the different HPCPs. At the end of the initial 4 months (period 1), subjects were switched to the other phase for a subsequent 4 months (period 2). Of 16 subjects enrolled and randomized, 3 withdrew. For one subject, no data were collected; a second dropped out midway after beginning phase 1; and a third withdrew after randomization after starting a course of antibiotics. The samples that were obtained were included in microbiome and metabolic analysis.

### Data collection.

Demographic information, including age, gender, and race/ethnicity, was collected (Table 2). Weight (in kilograms) was measured using a designated study scale unless otherwise noted. Blood, stool, gingival plaque, urine, and weight measures were obtained at baseline and at regular intervals throughout the study period. Samples were obtained following methods from the *Manual of Procedures for the Human Microbiome Project* (43). For gingival plaque samples, a toothpick was scraped along the gum line before cleaning teeth or eating, using the same molar and incisor throughout the study. Stool samples were obtained with a stool hat; a walnut-sized sample was placed in a vial using a spatula. Blood (24 ml) was collected via venipuncture by a trained phlebotomist.

**FIG 4:**
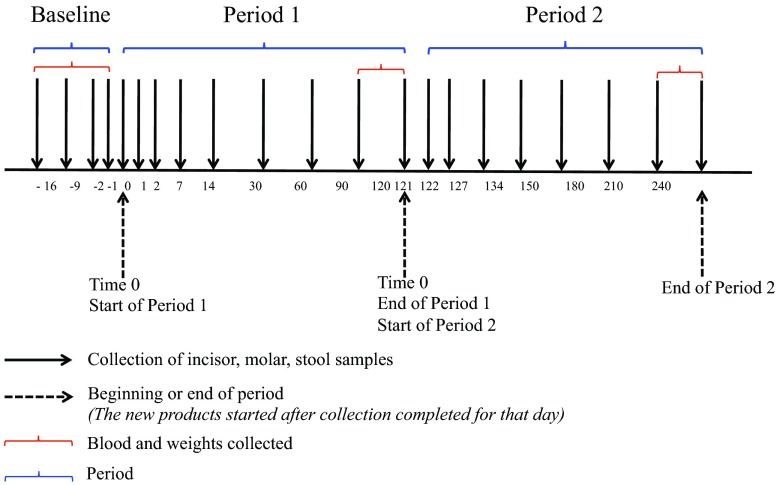
Sampling schedule for a participant. Black arrows indicate when incisor, molar, or stool samples were collected. Dashed arrows indicate the beginning and end of each period. Period data are shown in blue brackets; the time periods when blood and weight were collected are shown in red brackets.

Samples were collected following the sampling scheme illustrated in Fig. 4. In brief, stool and gingival plaque samples were collected prior to the use of study products at 14, 7, 2, and 1 days before beginning period 1 (“Baseline”). In period 1 and period 2, samples were collected on days 0, 1, 2, 7, 14, 30, 60, 90, and 120. Blood and weight values were collected at baseline and at the ends of both period 1 (day 120) and period 2 (day 240). Urine samples were collected intermittently throughout each period for a total of five samples per period.

### Blood and urine processing.

Blood samples obtained at the project laboratory were centrifuged, and the plasma was divided into aliquots. A 6-ml portion was frozen and later sent to the Stanford Clinical Laboratory and tested for total and free testosterone, triglycerides, thyroid-stimulating hormone, erythrocyte sedimentation rate, creatinine, and glucose. A second aliquot was passed through a 0.22-µm-pore-size filter and stored at −80°C. Aliquots were then shipped to Eve Technologies (Calgary, Alberta, Canada), where they were tested for 18 biomarkers of obesity, inflammation, and diabetes using the Discovery multiplex assays (diabetes and obesity panels) (Table 1; see Table S1 in the supplemental material). Urine was transferred from collection containers to 15-ml polypropylene tubes before being frozen. Urinary triclosan levels were measured with liquid chromatography-mass spectrometry with liquid-liquid extraction using ethyl acetate (see Text S1 in the supplemental material). Stable isotope-labeled triclosan (^13^C_12[CA1]_; Cambridge Isotope Laboratory, Tewksbury, MA) (99%) served as the internal standard, and blank urine from subjects with no to minimal exposure to triclosan was used as the sample matrix for calibration curve standards. Levels of free and glucuronidated triclosan were determined from their respective peaks and summed for total triclosan levels.

10.1128/mSphere.00056-15.2 Text S1  Supplemental methods: triclosan level detection. Download Text S1, DOCX file, 0.01 MB. Copyright © 2016 Poole et al. 2016 Poole et al. This content is distributed under the terms of the Creative Commons Attribution 4.0 International license.

### Statistical analysis of weight and biomarkers.

The distributions of weights and serum biomarker values at the end of each of the periods (baseline, period 1, and period 2) were visualized and explored for trends. Data from marker analyses run in duplicate were averaged. The differences in values across each period were compared using paired *t* tests or Wilcoxon signed-rank tests as appropriate. To account for multiple-hypothesis testing, Bonferroni correction was employed to determine statistical significance.

We assessed change in weight from the beginning and end of each period. The changes in differences across periods (i.e., gains or losses in each period) were compared using the McNemar test. The odds ratio (OR) and associated 95% confidence interval were then calculated.

Samples were measured in triplicate for urine triclosan measurements, and the means were used in analyses. When sample data were out of range for the detection assay, they were converted to the lower or upper bound. All means were divided by 10 to account for the initial sample concentration (see Text S1 in the supplemental material). Final values were converted from femtomoles per milliliter to picograms per milliliter. Median values of triclosan data were compared between phases using the Wilcoxon signed-rank test.

### Plaque and stool microbiome analyses.

Gingival and stool samples were placed in provided collection containers, immediately frozen by subjects in their home freezers, transported regularly to the laboratory in freezer packs, and frozen at −80°C. Bulk DNA was isolated from ~0.1 grams of stool using a PowerSoil DNA isolation kit as described by the manufacturer (MoBio Laboratories Ltd., Carlsbad, CA) and a bead beater set on high for 2 min (44, 45). DNA was similarly extracted from the toothpicks used to scrape molar and incisor gingival plaque. The V4 variable region of 16S rRNA genes was amplified via PCR using barcoded primers (515F-806R) (46). Duplicate PCRs were performed using a previously published PCR protocol (47), with the following modifications: for plaque samples, the number of cycles was 30; for stool samples, the number of cycles was 25 and the primer concentration was reduced to 0.05 µM. Amplicons from the duplicate reaction mixtures were combined, purified using MagBind EZPure (Omega Biotech), and quantified using a Quant-iT picogreen double-stranded DNA (dsDNA) assay kit (Invitrogen). Purified amplicons were pooled equimolarly and sequenced (2 × 250 bp) using the MiSeq System at the Cornell University Biotechnology Resource Center Genomics Facility (47).

Gingival plaque sample sequence data and stool sample sequence data were analyzed separately. Mated paired-end sequences were merged, quality filtered, and analyzed using the QIIME software package 1.9.0 (48). We performed open-reference operational taxonomic unit (OUT) picking at 97% identity using the August 2013 Greengenes database for the initial closed reference step. OTU abundances for each sample type (molar gingival plaque, incisor gingival plaque, or stool) were normalized in QIIME using the cumulative sum scaling (CSS) transformation to account for differences in sequencing depth between samples, and then OTUs with the same taxonomy were combined at the genus level into taxa (49).

To ensure that the treatment for each phase had enough time to impact the microbiome, only sequence samples collected during the second half of each phase (i.e., after day 60 of each phase) were used in the analysis (Fig. 4). This time scale was determined because urine and plasma levels of TCS in humans were previously reported to return to baseline 8 days after the exposure ended (4, 50). Though TCS’s effects on the human microbiome have not been extensively studied, it has been observed that the presence of TCS immediately alters the minnow microbiome, which then takes 2 weeks to recover after cessation of TCS exposure (51). For alpha diversity assessment, rarefied OTU tables were generated with subsampling of between 10 and 39,800 sequences in steps of 100 sequences, performing 10 iterations at each sampling depth. Alpha diversity assessment using multiple metrics (Faith’s phylogenetic diversity, Chao 1, and Observed Species) was then performed on the rarefied tables (52, 53).

To search for taxa that were differentially abundant in the two phases, nonrarified data were CSS normalized, and sequences belonging to OTUs with a shared taxonomy were combined into “taxa” (i.e., collapsed taxonomies). Taxa that were not shared by at least 40% of the individuals were excluded from the analysis. Using the lme4 package in R, a linear mixed-effects model was applied, where the square root-transformed abundances of the taxa were the response variables, starting period and phase were fixed effects, and random terms were included to account for repeat measures from the same subject within and across phases. The false-discovery rate of 0.05 was controlled for using the Benjamini-Hochberg procedure.

### Nucleotide sequence accession numbers.

Sequences have been deposited in the European Nucleotide Archive under accession no. PRJEB12496 (oral microbiota) and PRJEB12499 (stool microbiota).
