# Supplementary material for: Crossover Control Study of the Effect of Personal Care Products Containing Triclosan on the Microbiome
Source: mSphere. 2016 May 18;1(3):e00056-15. doi: 10.1128/mSphere.00056-15 (PMC4888890; doi:10.1128/mSphere.00056-15)
Supplement: Table S1 [file sph003162083st2.docx]

**Supplemental Table 1: Median values of remaining obesity and diabetes markers by phase**

|  | Baseline median (Q1-Q3) | Median at end of TCS phase | Median at end of nTCS phase | p (1) |
| --- | --- | --- | --- | --- |
| Adiponectin (µg/mL) * | 6.3(3.7-9.4) | 6.6 | 7.4 | 0.24 |
| Adipsin (µg/mL) * | 0.4(0.1-1.1) | 0.8 | 0.7 | 0.38 |
| C-peptide | 635 (378-748) | 315 | 362 | 0.4 |
| Creatinine (mg/dL) | 0.7(0.6-0.7) | 0.8 | 0.8 | 0.66 |
| ESR(mm/hr) | 6 (2-13) | 6 | 8 | 0.26 |
| Free T4 (ng/dL) | 1.0 (0.9-1) | 1 | 1 | + |
| Ghrelin | 708 (340-970) | 553 | 387 | 0.14 |
| GLP1 | 150 (116-171) | 111 | 117 | 0.75 |
| Glucagon | 165 (130-193) | 135 | 143 | 0.66 |
| Glucose (mg/dL) | 87(79-96) | 88 | 89 | 0.22 |
| IL-10 | 4.3 (2.5-4.5) | 3.7 | 3.1 | 0.73 |
| IL-6 | 7 (7-7.5) | 7 | 7 | 0.59 |
| MCP-1 | 91.5 (71.2-103.3) | 96 | 95 | 0.79 |
| Resistin (ng/mL) * | 2.8 (2.3-3.4) | 3 | 3.2 | 0.49 |
| Serpin E1 (ng/mL) * | 4.4(3.3-7.2) | 6.2 | 7.9 | 0.48 |
| TNF alpha | 2.4 (0.4-3.1) | 0.41 | 1.1 | + |
| Total Testosterone (ng/dL) | 27 (23-330) | 26 | 31 | 0.13 |
| Visfatin (ng/mL) | 2.0 (0.6-3.1) | 0.8 | 0.9 | + |
| ESR: erythrocyte sedimentation rate; GLP1: Glucagon-like peptide-1; IL: Interleukin; MCP1: Monocyte chemotactic protein-1; T4: thyroxine; TNF: Tumor necrosis factor;  All values in pg/mL unless as noted  (1) The difference in values between end of each phase and the beginning of that phase was calculated The two differences were then compared with the Wilcoxon-signed rank test  + Too few samples returned valid values  * Repeated measures | | | | |
