# Supplementary material for: Crossover Control Study of the Effect of Personal Care Products Containing Triclosan on the Microbiome
Source: mSphere. 2016 May 18;1(3):e00056-15. doi: 10.1128/mSphere.00056-15 (PMC4888890; doi:10.1128/mSphere.00056-15)
Supplement: Text S1 [file sph003162083s1.docx]

**Supplemental Materials:**

**Methods:**

**Triclosan level detection:**

Urinary triclosan (TC) levels were measured with liquid chromatography-mass spectrometry with liquid-liquid extraction using ethyl acetate. Stable isotope labeled triclosan (^13^C_12[CA1]_ [KKM2] [KKM3] [KKM4] [KKM5] , 99%, Cambridge Isotope Laboratory) served as the internal standard (TC IS) and blank urine from subjects with no to minimal exposure to TC was used as sample matrix for calibration curve standards. Additionally, triclosan glucuronide (TC-G, Sigma-Aldrich) standard was used for monitoring conjugated TC vs. free TC.

Because the majority of TC in urine is present in the glucuronide form, each sample underwent enzymatic hydrolysis to convert TC-G to TC. 4-methylumbelliferone (4MU) and 4-methylumbelliferone glucuronide (4MU-G[CA6]) were used to monitor the efficiency of the deglucuronidation reaction. To 1 mL of urine, 10 µL of 20 µL M TC IS stock solution and 10 µL L of 50 µM 4MU-G were added, followed by 800 µL of 1 M ammonium acetate and 20 µL L of deglucuronidase solution (1200units/mL, 1M ammonium acetate, pH 5).[CA7] Samples were incubated for 2 hours at 37°C to allow hydrolysis of glucoronide conjugates. The enzymatic activity was then stopped with 300 µL L of 1 M formic acid. The hydrolyzed samples were treated with 1.5 mL of ethyl acetate, briefly vortexes, and shaken for 5 min.[CA8] The upper organic phase was collected and dried under a stream of nitrogen gas. Samples were reconstituted in x µL L of 20% methanol and transferred to autosampler vials.

The LC-MS/MS analysis was performed on a TSQ Vantage triple quadrupole mass spectrometer coupled with an Accela 1250 HPLC (Thermo Fisher Scientific). Injection volume was 10 µL. Reversed phase separation was carried out on a Kinetex C18 column (50 mm x 2.1 mm ID, 2.6 um particle size, Phenomenex). Mobile Phase A was water, and mobile phase B was methanol; flow rate was 350 µL L per minute. The gradient was as follows: 0 min. (20% B), 2.5 min. (98% B), 4 min. (98% B), 4.5 min (20% B) and 6 min (20% B). The mass spectrometer was operated in negative APCI mode, with selected reaction monitoring (SRM). Three SRM transitions were used for each TC and TC IS: 250.9 > 159.1, 187.0, 214.9 and, 263.07 > 169.0, 197.9, 226.9, respectively. The calibration curve was linear from 1 to 40,000 fmol/µL, and the lower limit of quantitation (LLOQ) was around 10 fmol/ µL L of TC in extracted urine.

Samples were measured in triplicate. All results were divided by 10 to account for concentration in processing. Triclosan and glucuronidated triclosan levels were determined from their respective peaks on mass spectroscopy and then summed.
